# Effect of Fentanyl Infusion on Heart Rate Variability and Anaesthetic Requirements in Isoflurane-Anaesthetized Horses

**DOI:** 10.3390/ani11102922

**Published:** 2021-10-09

**Authors:** Petra Dmitrović, Jana Vanaga, Julien Dupont, Thierry Franck, Alexandra Gougnard, Johann Detilleux, Liga Kovalcuka, Alexandra Salciccia, Didier Serteyn, Charlotte Sandersen

**Affiliations:** 1 Faculty of Veterinary Medicine, University of Liège, 4000 Liège, Belgium; petra.dmitrovic@uliege.be (P.D.); julien.dupont@uliege.be (J.D.); T.Franck@uliege.be (T.F.); alexandra.gougnard@uliege.be (A.G.); jdetilleux@uliege.be (J.D.); alexandra.salciccia@uliege.be (A.S.); didier.serteyn@uliege.be (D.S.); 2 Faculty of Veterinary Medicine, Latvia University of Life Sciences and Technologies, LV-3004 Jelgava, Latvia; jana.vanaga@gmail.com (J.V.); kovalcuka@gmail.com (L.K.)

**Keywords:** fentanyl, isoflurane, anaesthesia, horse, PTA

## Abstract

**Simple Summary:**

Historically, the use of opioids in equine anaesthesia has been limited due to many reported adverse effects. Even though more research is being conducted, there is conflicting evidence on the efficacy of opioids and their behavioural effects in horses. The aim of this study was to investigate the effect of the synthetic opioid fentanyl on various parameters measured during general anaesthesia, as well as to determine its possible sparing-effect on inhalant anaesthetic.

**Abstract:**

Controversy continues to surround the use of opioids in equine anaesthesia, with variable effects reported. This blinded clinical study aimed to investigate the influence of a low-dose fentanyl continuous rate infusion (CRI) on isoflurane requirements, parasympathetic tone activity (PTA), and anaesthetic parameters in horses during general anaesthesia. All of the twenty-two horses included in the research underwent a standard anaesthetic protocol. Eleven horses in the fentanyl group (Group F) received a loading dose of fentanyl at 6 µg/kg, followed by a CRI of 0.1 µg/kg/min during anaesthesia. A further 11 horses in the control group (Group C) received equivalent volumes of normal saline. Anaesthetic parameters and PTA index were recorded during anaesthesia. The achieved mean fentanyl plasma concentration was 6.2 ± 0.83 ng/mL. No statistically significant differences between groups were found in isoflurane requirements, MAP values, and mean dobutamine requirements. However, horses in Group F required a significantly lower dose of additional ketamine to maintain a sufficient depth of anaesthesia. Significantly higher PTA values were found in the fentanyl group. Further research is warranted to determine the limitations of PTA monitoring, and the influence of various anaesthetics on its values.

## 1. Introduction

Opioids are commonly used in numerous animal species, often as a part of balanced anaesthesia protocols, where their analgesic and sedative effects reduce the requirement for other anaesthetic agents [1]. However, the use of opioids in horses has been somewhat controversial and often limited, due to reported undesirable side effects [2,3,4,5,6,7]. Horses have been known to respond to opioids with excitement and increased locomotor activity (probably a consequence of central nervous system excitation), and a decrease in gastrointestinal motility. It seems that the magnitude of these side effects is related to the drug and dose administered, as well as to an individual sensibility. A recent study showed that increased locomotor activity is probably related to G57C μ-opioid receptor polymorphism, expressed in heterozygous animals [8]. Furthermore, there seems to be a narrow therapeutic margin between the doses required to produce analgesia and excitation [1], which prompts further research to find precise doses for each opioid receptor agonist. 

Fentanyl is a synthetic, short-acting, full µ-opioid receptor agonist. It is one of the most commonly used drugs to treat moderate and severe pain in humans and many other animal species [9]. Multiple studies have evaluated the effect of fentanyl during general anaesthesia in horses, and although a dose-dependent minimal alveolar concentration (MAC) sparing effect is described in humans [10], pigs [11], sheep [12] and dogs [13], this seems to be less pronounced in horses [14,15].

The evaluation of nociception during anaesthesia can be challenging, and it is normally based on cardiovascular variables such as an increase in heart rate and blood pressure, change of respiratory pattern, or movements of the animal. However, changes in these variables are not specific to nociception exclusively [16]. Having a tool which would continuously monitor the nociception/antinociception balance during anaesthesia and provide early detection of potential haemodynamic reactivity would help the anaesthetist provide optimal analgesia [16].

As modern anaesthesia strives to find the minimal effective dose of drugs, to avoid both analgesic under- and over-dosing [17,18], such devices have recently been developed and tested. In human medicine, the Analgesia Nociception Index (ANI) is being used not only for detection of nociception during anaesthesia, but also to predict post-operative pain during recovery from anaesthesia [16,19]. The Parasympathetic Tone Activity (PTA) monitor is a version of ANI monitor, the first of a kind in veterinary medicine. The operating principle of this monitor has been described previously [16,17,20,21]. In brief, it detects heart rate variability (HRV) and gives an ‘immediate’ and ‘averaged’ index. The index is quantifiable from 0 to 100 and is continuously displayed. High PTA values indicate a high parasympathetic tone, whilst low values reflect a low tone [16,22]. These values therefore represent the sympatho-vagal balance and may indicate an analgesia/nociception imbalance when measured during nociceptive stimulation [20]. The PTA monitor may help to optimize patient status during anaesthesia and guide the decision of additional analgesia administration. It may also help to evaluate the effects of a particular analgesic drug used during surgery [16,21].

This study aimed to investigate the effect of fentanyl on isoflurane requirements and establish its effect on PTA measurements during common surgical procedures carried out in isoflurane-anaesthetized horses. We hypothesized that with the addition of fentanyl, a reduction in isoflurane requirements would be detected, as well as an effect on PTA values. The authors wanted to investigate whether, at the achieved fentanyl plasma concentration, the PTA index would rise because of better analgesia and less stress from nociception, or decrease due to a sympathomimetic effect previously described in horses [15].

## 2. Materials and Methods

### 2.1. Animals

Based on previous unpublished observations of a mean end-tidal isoflurane (EtIso) concentration of 1.2 ± 0.16% and 1.0 ± 0.16% in control and fentanyl-treated horses, respectively, and aiming to achieve a power of 80% and a level of significance of 5%, a sample size of 11 horses per group was calculated. This clinical study was approved by the Animal Ethics Committee of University of Liège (permit number 1989) and written informed owner consent was obtained for all the animals included.

A total of 22 client-owned adult horses of different breeds, admitted for various surgical procedures, were recruited for this blinded study. Horses were included in the study if their American Society of Anesthesiologists (ASA) physical status was I-III based on medical history, clinical examination and routine pre-anaesthetic blood analysis. Horses given a higher ASA status (IV–V), under 2 years of age, or weighing less than 200 kg were not included. An additional exclusion criterion was a total time under anaesthesia shorter than 90 min.

Horses were randomly assigned to one of the two groups by drawing a paper from an envelope containing an equal number of papers marked F (fentanyl group) and C (control group). Then, a horse of similar weight and age anaesthetized for a similar procedure was recruited and assigned to the opposite treatment group. The anaesthetist was unaware of the treatment assigned. Anaesthesia was equally distributed between the two anaesthetists (J.Du. or A.G.) in a random order.

### 2.2. Anaesthesia and Instrumentation

Horses admitted for scheduled surgeries were fasted for 12 h, with free access to water, while those admitted for emergency colic surgery had variable fasting times. All horses had a 14 G catheter placed in the jugular vein for drug and fluid administration. They were premedicated with acepromazine 0.1 mg/kg IM (Placivet^®^, Kela, Hoogstraten, Belgium), except if they were admitted for colic surgery. All horses were sedated with xylazine 0.6 mg/kg IV (Proxylaz^®^, Prodivet, Eynatten, Belgium) and anaesthesia was induced with midazolam 0.06 mg/kg (Midazolam Mylan^®^, Mylan, Hoeilaart, Belguim) and ketamine 2.2 mg/kg IV (Ketamidor^®^, Ecuphar, Oostkamp, Belgium). After induction, horses were intubated orotracheally with an appropriate size cuffed silicone endotracheal tube and positioned on a padded surgical table. They were then transferred to the operating theatre and connected to the Tafonius large animal anaesthesia machine (Vetronic, Abbotskerswell, UK) via a large animal Y-piece breathing circuit. General anaesthesia was maintained with isoflurane (IsoFlo^®^, Abbott, Maidenhead, UK) delivered in oxygen using intermittent positive pressure ventilation (IPPV). Lactated Ringer’s solution (Vetivex^®^, Dechra, The Netherlands) was infused intravenously to all horses, at a rate of 2–4 mL/kg/h during anaesthesia. A urinary catheter was placed, and the bladder was drained continuously throughout anaesthesia to minimize bladder distention during surgery and before recovery.

Tafonius’s integrated monitoring equipment was used for ECG, invasive blood pressure, pulse oximetry, capnography, airway pressure and anaesthetic agent concentration measurement. A standard 3-lead ECG was used, with crocodile clamps attached to the neck and both sides of the thorax at the level of the heart. The pulse oximeter probe was placed on the tip of the tongue. Respiratory rate and tidal volume were initially set at 10 mL/kg and 10 respirations per minutes and then adapted to maintain end tidal CO_2_ (EtCO_2_) between 35 and 45 mmHg, and peak inspiratory pressure (PIP) at 25–35 cmH_2_O. Facial artery was percutaneously catheterized with a 20 G catheter which was then connected to a transducer levelled with the sternum. Systolic arterial blood pressure (SAP), diastolic arterial blood pressure (DAP) and mean arterial blood pressure (MAP) were recorded. If a decrease in MAP below 65 mmHg occurred, dobutamine (Dobutrexmylan 250 mg/20 mL, Mylan, Hoeilaart, Belgium) was infused by a syringe perfusion pump (Fresenius Vial Infusion Technology, Pilot A2 infusion pump, Brezins, France), titrated to maintain MAP values between 65 and 90 mmHg. Every 20 min a blood sample was drawn from the catheter for arterial blood gas analysis (Cobas^®^ b 123 POC blood gas analyser, Roche Diagnostics, Brussels, Belgium). Hearth rate, rhythm, and parasympathetic tone activity (PTA) were measured and recorded by a PTA Monitor (Mdoloris Medical System, Loos, France) connected to the analogue output of the Tafonius’s multiparameter monitor. In addition to the automatic recording of monitoring devices, all data were manually recorded by the anaesthetist every five minutes during anaesthesia.

### 2.3. Determination of Isoflurane Requirement and Fentanyl Administration

At the beginning of general anaesthesia, horses were maintained with isoflurane vaporized in oxygen, delivered at a rate of 6–8 L/min to achieve optimal anaesthetic depth as evidenced by the following indicators: eyes rotated into a rostroventral position, the presence of a weak palpebral reflex and absence of nystagmus [23]. After optimal anaesthetic depth was achieved, EtIso concentration was maintained constant for 15 min. This period is referred to as ‘before treatment. Due to individual variations in time that passed from the administration of anaesthesia to the achievement of the optimal anaesthetic depth, the period ‘before treatment’ had a varying duration, with a mean time of 28 min the next step was begun, referred to as ‘the treatment’. In this second period, horses in Group F received a bolus of 6 µg/kg of fentanyl (Fentanyl®-Janssen, 50 µg /mL, Janssen, Belgium) intravenously, followed by a constant rate infusion (CRI) of 0.1 µg/kg/min [15]. Horses in Group C received saline at the volume corresponding to the volume of fentanyl loading dose and the CRI, to ensure the anaesthetist was unaware of the treatment. The drugs were prepared by a veterinarian not involved in the anaesthetic care of the horse.

For the next 20 min, the EtIso concentration was maintained constant, after which it was gradually reduced every 15 min in increments of 0.1%, to the lowest concentration providing adequate anaesthetic depth based on observation of the patient. Anaesthetic depth was estimated insufficient if a brisk palpebral reflex, nystagmus, spontaneous movement or sudden changes of cardiovascular variables were observed. In case of such events, a bolus of ketamine was administered at 0.3 mg/kg IV and EtIso was increased to the last value which maintained adequate anaesthetic depth, and marked as the lowest isoflurane concentration achieved in that horse (Iso_low_). The CRI of fentanyl or saline was stopped approximately 30 min before the expected end of anaesthesia, as communicated with the surgeon, to avoid the possible excitatory effect of fentanyl during recovery.

### 2.4. Determination of Fentanyl Plasma Concentration

Samples were obtained from the arterial catheter one minute after the end of bolus administration (fentanyl or saline), followed by sampling every 20 min until the end of anaesthesia. The number of samples taken varied according to length of anaesthesia. Blood was collected with a syringe and transferred into lithium heparin blood collection tubes. After 10 min of centrifugation at 4000 rpm, plasma was separated and stored at −20 °C until analysis was performed. Fentanyl concentrations in plasma were determined by enzyme-linked immunosorbent assay (ELISA), as previously described [24]. The Fentanyl Racing Elisa kit from Neogen (Rochdale, UK) was used. This direct competitive ELISA operates on the basis of competition between horseradish peroxidase (HRP) enzyme conjugate and the fentanyl in the sample. The richer the plasma is in fentanyl, the less the fentanyl conjugate binds to the antibodies. Consequently, the extent of colour development is inversely proportional to the amount of fentanyl in the sample.

### 2.5. Evaluation of PTA

The PTA index was measured with a PTA monitor (Mdoloris Medical System, Loos, France), using a lead II ECG signal obtained with a standard 3-lead ECG from the multiparameter monitor (Tafonius, Vetronic, Abbotskerswell, UK). The PTA monitor was calibrated for use in horses. The PTA values (0–100) were measured continuously and recorded every five minutes. Both immediate and averaged values were recorded, but only the averaged values were used in statistical analysis.

### 2.6. Recovery Assessment

All horses were sedated with xylazine at the beginning of recovery (initial dose 0.2 mg/kg IV). The dose was repeated according to individual needs until the nystagmus stopped and the horse was ready to stand up. All animals recovered with the assistance of head and tail ropes. The quality of recovery was assessed by the anaesthetist using a simple numerical scale from 1 to 5 (1 being the best recovery and 5 the worst).

### 2.7. Statistical Analysis

Data analysis was performed with a commercial statistics software Statistical Analysis System, SAS Inc., Cary, NC, USA). Normality of data distribution was tested with Shapiro–Wilk test. Body weight, anaesthesia length, RR, HR, MAP, dobutamine requirements, V_t_, PIP, EtIso concentration, PTA, ketamine dose, PaO_2_, PaCO_2_ and xylazine dose were all tested with an independent t-test to compare the groups and with a paired t-test to compare data within one group before and during treatment. Significance was set at *p* < 0.05. Data are reported as mean ± standard deviation. The recovery scores were compared using a Mann–Whitney U-test and are presented as a mean score, as well as the range.

## 3. Results

Eleven horses were recruited in the fentanyl group. Their body weight ranged from 210 to 645 kg (mean 455 ± 137 kg) and their age ranged from 2 to 23 years (mean 9 ± 7 years). They underwent the following procedures: laparotomy for colic surgery (*n* = 8), arthroscopy (*n* = 1), ophthalmic procedure (*n* = 1), tenoscopy (*n* = 1). The mean ± SD fentanyl plasma concentration was 6.2 ± 0.83 ng/mL during administration of fentanyl. Eleven matched control horses with a body weight of 450 to 580 kg (mean 513 ± 43 kg) and an age of 4 to 25 years (mean 11 ± 7) underwent laparotomy for colic surgery (*n* = 6), arthroscopy (*n* = 1), castration (*n* = 1), ophthalmic procedures (*n* = 2) and wound debridement (*n* = 1). Data are summarized in Table 1.

**Figure 1:**
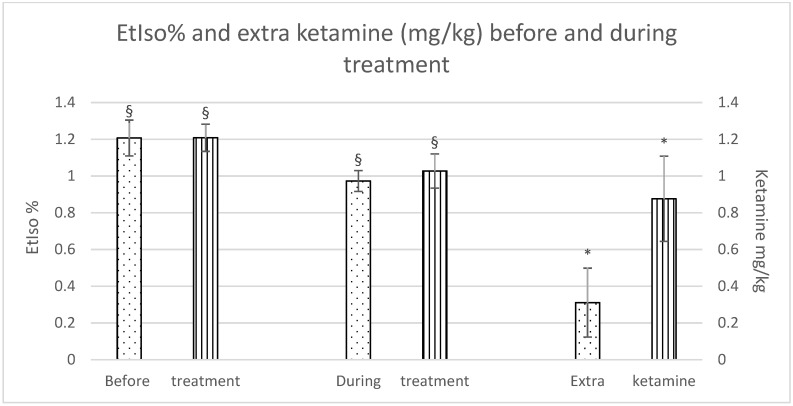
EtIso% and extra ketamine (mg/kg) before and during treatment (fentanyl dots, control stripes). *p* < 0.05. * = significantly different between groups; § = significantly different before and during treatment.

Although there were no significant differences in isoflurane reduction between the groups, a significant difference was observed within each group during the treatment as opposed to before the treatment was started. Furthermore, horses in the Group C required a significantly higher dose of extra ketamine to maintain an adequate depth of anaesthesia (Group F: 0.3 ± 0.4 mg/kg; Group C: 0.9 ± 0.5 mg/kg), as shown in Figure 1.

**Figure 2:**
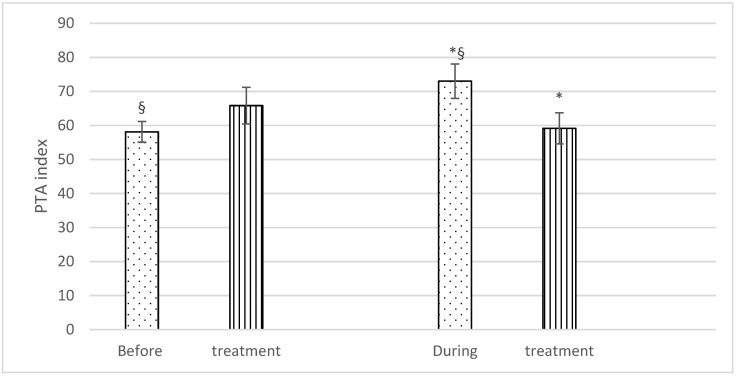
Mean PTA (± SD) of fentanyl (dots) and control (stripes) group, before and during treatment. *p* < 0.05. * = significantly different between groups; § = significantly different before and during treatment.

With regard to PTA, no significant differences were found before treatment (Group F: 58.1 ± 6.4; Group C: 65.8 ± 11.4), but significantly higher PTA values were recorded during the treatment in Group F (73 ± 10.6) than in Group C (59.1 ± 9.6). There was also a statistically significant difference in PTA values in Group F horses before and during treatment (58.1 ± 6.4 and 73 ± 10.6), respectively) (Figure 2).

No significant difference was found when the recovery scores were compared between the groups. Group F scored a mean of 1.8 (range 1–4), and Group C, 2.1 (range 1–5).

## 4. Discussion

The results of this study show that in both groups, the mean EtIso was lowered over the anaesthesia period. Horses who received fentanyl needed significantly less ketamine to maintain sufficient depth of anaesthesia than the control horses. This study also detected a significant difference in the PTA index between the two groups, with higher values in Group F, indicative of a higher parasympathetic tone.

In this study, we chose to administer a loading dose of fentanyl of 6 µg/kg, followed by a CRI of 0.1 µg/kg/min, achieving a plasma concentration (C_p_) of 6.2 ± 0.83 ng/mL. Similar doses were used by Ohta et al. [15] during sevoflurane anaesthesia, with an achieved C_p_ ranging from 6.12 ± 0.88 to 7.78 ± 1.12 ng/mL. Some authors achieved much higher C_p_ (13.31 ng/mL) with a lower loading dose, followed by a similar rate CRI [14]. Pharmacokinetics can be influenced by age, sex, breed, other drugs administered, and the physical condition of the animal. Other factors that could cause such discrepancies include differences in premedication protocols, different inhalant anaesthetic agents used (isoflurane vs. sevoflurane), and total anaesthesia time (mean anaesthesia time 464 min [14], 118 min [15] compared to 132 min in our study). Thomasy et al. [25] also demonstrated a lower clearance of fentanyl in healthy, isoflurane-anaesthetized horses compared to conscious horses. This might be explained by a decrease in renal blood flow during general anaesthesia, as the primary metabolite of fentanyl is excreted in urine. The achieved Cp depends on the dose and route of administration, as well as on speed of injection and the timing of blood sampling for Cp quantification [4,6,14,15,26,27,28]. An individual variation between targeted and achieved Cp has been observed at various doses [26,27,29]. While for this study we used ELISA using arterial blood samples, reports by Thomasy et al. [14] and Ohta et al. [15] determined the C_p_ from venous blood, using liquid chromatography–mass spectrometry. To the authors’ knowledge, the arterio-venous C_p_ difference after IV administration of fentanyl in horses is unknown, as well as its clinical importance. In human medicine, there is proof that arterial sampling of intranasally administered fentanyl is better to determine the onset of analgesia, while venous sampling failed to detect its rapid onset [30]. An effective analgesic Cp of fentanyl has recently been established in horses. It appears that a value of ≥ 6.1–6.8 ng/mL provides an anti-nociceptive effect lasting 10–30 min against a thermal stimulus [31]. This concentration is remarkably higher than the analgesic plasma concentration reported in cats (>1.07 ng/mL) and dogs (>0.95 ng/mL) [32,33], probably depicting a weaker analgesic effect of opioids in horses.

Our study found no statistically significant difference in the mean MAP or dobutamine requirements between groups. Unfortunately, the anaesthetist did not precisely titrate the dobutamine rate to effect. A more rigorous control of dobutamine consumption would have allowed us to better determine and separate the effects of dobutamine and fentanyl. Our study therefore failed to support the results of some authors [15] who observed less dobutamine requirement when fentanyl was infused during anaesthesia, as it provided better haemodynamic stability in comparison with sevoflurane alone.

In this study, the administration of fentanyl resulted in an 18% reduction in isoflurane requirement during the course of anaesthesia in Group F. Because of a negligible difference in isoflurane requirement between the two groups (18% in Group F and 15.2% in Group C), we consider the results to demonstrate the isoflurane-sparing effect of fentanyl and ketamine, respectively. Our results are in agreement with those of Thomasy et al. [14], where a MAC-sparing effect of 18% was detected with the use of fentanyl. However, the reduction in our study was not statistically significant, whereas Thomasy et al. [14] found this isoflurane-sparing effect to be statistically significant, although clinically irrelevant. In a study by Ohta et al. [15], fentanyl infusion was used in sevoflurane-anaesthetized horses undergoing orthopaedic surgery, and a MAC reduction of 13% was recorded. Conversely, in an experimental study, Kynch et al. [4] found no MAC-sparing effect of fentanyl in isoflurane-anaesthetized horses. This is in contrast to humans [10] or other animal species [11,12,13,34,35], where reductions of up to 82% and 22.6–56.6%, respectively, have been shown. One may therefore question the benefits of this drug in equine general anaesthesia, as in this species, balanced anaesthesia aims particularly at reducing cardiovascular depression caused by inhalant anaesthetics. Although in the present study, we could not demonstrate a clinically relevant reduction in isoflurane requirement, nor a cardiovascular-sparing effect, we found a statistically significant difference between the two groups in the total amount of ketamine used. The design of the study and the fact that it included client-owned horses restricted us from relying on isoflurane to increase anaesthetic depth due to its slow onset, and therefore, ketamine was used. Horses in Group C needed 3 times more ketamine ‘rescue’ boluses. This could be a consequence of achieving a reduction in nociception and a more balanced anaesthesia with fentanyl, as the mean Cp achieved (6.2 ng/mL) could be considered an analgesic concentration in horses [31]. A reduced MAC of inhalant anaesthetic agents can be achieved with opioids in small animals, accompanied by improved hemodynamics [36,37].

The simple scoring system used in this study to assess the quality of recovery did not reveal any differences between groups. Furthermore, the only horse that recovered poorly (scoring 5) did not receive fentanyl treatment. However, Kynch et al. [4] described undesirable behaviour during recovery in some horses, which led those authors not to recommend the use of fentanyl infusion during isoflurane anaesthesia in horses. Opioids tend to induce sympathetic and central nervous stimulation in horses, contrary to a depressing effect seen in most species [38]. This can be explained by a different distribution of opioid receptors in horses [39]. A more sophisticated, validated scoring system could have potentially revealed some differences in the quality of recoveries between the two groups of horses investigated.

Finally, a significant difference was detected in the PTA values between the two groups during the treatment, with higher values in Group F. Furthermore, the PTA value was significantly higher in Group F during the fentanyl infusion, as compared to before treatment. Higher PTA is usually interpreted as a consequence of better antinociception [17], as parasympathetic stimulation prevails. A recent study [21] described that nociception is detected more quickly with a PTA monitor when lower-intensity nociceptive stimulation is applied. However, with high-intensity nociceptive stimulation, the monitor failed to detect the changes quickly enough, as cardiovascular changes were observed before a PTA response. Nevertheless, we should not exclude a higher PTA index as a consequence of the effect of fentanyl on HRV. One should be aware that the HRV does not represent an absolute level of autonomic activity, it only depicts the influence of parasympathetic and sympathetic systems on sinoatrial node and therefore heart rate [40]. Fentanyl has been known to have a sympatholytic effect, with a trend toward vagal activation in most species [40]. On the other hand, one study demonstrated a dose-dependent effect of fentanyl on heart rate and MAP values, suggesting a sympathomimetic effect of this drug in horses [15]. However, plasma concentrations were higher than those measured in our study, in which we did not observe this sympathomimetic effect. Unfortunately, information regarding the influence of different anaesthetic drugs on PTA index in animals is lacking; most of the information is extrapolated from human medicine, which might lead to faulty conclusions.

One of the limitations of assessing analgesic effect in this study was the difference between the types of surgeries investigated. The animals therefore experienced different types and intensities of pain, and different degrees of surgical insult. The timing of the painful event was not recorded during the study and could, therefore, not be related to the changes of the PTA values. A future study should focus on strict case selection with more standardized surgical procedures and a recording of painful events during anaesthesia. Secondly, as colics were included in the study, a certain degree of pre-existing cardiovascular compromise was probably present. This could have interfered not only with PTA measurement, but also with the pharmacokinetics of the fentanyl. Thirdly, some horses received acepromazine in their premedication, which could also have affected their PTA values. Another limitation is the lack of validation of the PTA method in horses. Although an algorithm has been designed for use in horses, the method remains to be validated. Finally, using subjective assessment to evaluate the depth of anaesthesia makes the interpretation of results regarding isoflurane reduction more difficult. Subjectivity in the assessment of anaesthetic depth remains an important limitation, as two anaesthetists provided anaesthesia for horses in our studies. Unfortunately, no algorithm or flow-chart was used to reduce isoflurane concentration in this study. Previous studies [41,42] have shown that clear algorithms are useful to standardize the reduction in isoflurane administration, and this may help to avoid a too rapid reduction in inhalant anaesthetic and an abrupt change of anaesthetic depth. The influence of ketamine on PTA values in horses is unknown. A recent study in humans reported that a ketamine bolus of 0.5 mg/kg only induced a slight decrease in ANI values, without any statistical or clinical significance [43]. These results are important as they show that when used in low doses, ketamine does not exhibit an important sympathomimetic effect which could interfere with the measurement of parasympathetic tone, and therefore, possibly lead to the misinterpretation of ANI values. However, the authors cannot exclude the sympathomimetic effect of ketamine at the doses used in Group C, and therefore, this is a potential cause for significantly lower PTA values in this group.

## 5. Conclusions

Although much remains to be clarified, we conclude that fentanyl at the dose rates tested could be used as a component of a balanced anaesthesia protocol in horses. It resulted in a three-fold decrease in the requirement for additional ketamine boluses and a concomitant increase in PTA values in clinical cases anaesthetized with isoflurane. Although no isoflurane- or cardiovascular-sparing effects were found, fentanyl does appear to help balance general anaesthesia in horses. Further research is warranted to determine the limitations of PTA monitoring, and the influence of various anaesthetics on its values.

## Figures and Tables

**Table 1:** Mean values for pre- and intraoperative variables in fentanyl and control group.

Weight (kg)	Group F (C_p_ 6.2 ± 0.83 ng/mL)	Group C
	458.7 ± 130.5	513.2 ± 43.2
Duration of isoflurane administration (min)	131.8 ± 51.4	138.2 ± 47.2
Respiratory rate (rpm)	10.2 ± 1.3	9.9 ± 1.5
Tidal volume (mL/kg)	12.1 ± 1.6	11.1 ± 1.1
Peak inspiratory pressure (cmH_2_O)	23.7 ± 4.5	22.6 ± 7.2
Mean PaO_2_ (mmHg)	216.2 ± 128.3	204 ± 123.2
Mean PaCO_2_ (mmHg)	50.6 ± 4.3	51.8 ± 10.9
Mean HR before fentanyl or control treatment (bpm)	39.6 ± 7.5	42.3 ± 10.9
Mean HR during fentanyl or control treatment (bpm)	39.9 ± 7.1	41.9 ± 10.7
Mean EtIso (%) before fentanyl or control treatment	1.2 ± 0.2 ^§^	1.2 ± 0.2 ^§^
Lowest EtIso (%) during fentanyl or control treatment	1.0 ± 0.1 ^§^	1.0 ± 0.2 ^§^
EtIso reduction (%)	18.0 ± 13.0	15.2 ± 12.5
Mean MAP (mmHg) before fentanyl or control treatment	76.9 ± 15.1	81.1 ± 23.3
Mean MAP (mmHg) during fentanyl or control treatment	92.7 ± 17.3	84.7 ± 21.6
Total dobutamine (µg/kg)	54.1 ± 58.5	33.7 ± 28.7
Mean PTA before fentanyl or control treatment	58.1 ± 6.4 ^§^	65.8 ± 11.4
Mean PTA during fentanyl or control treatment	73 ± 10.6 *^,§^	59.1 ± 9.6 *
Extra ketamine (mg/kg)	0.3 ± 0.4 *	0.9 ± 0.5 *
Total dose of xylazine on recovery	0.4 ± 0.1	0.4 ± 0.2

Data given as mean ± standard deviation for 11 horses in the fentanyl group and 11 horses in the control group. *p* < 0.05. * = significantly different between groups; § = significantly different before treatment and during treatment. Rpm = respirations per minute; bpm = beats per minute.

## Data Availability

The data presented in this study are openly available in FigShare at doi:10.6084/m9.figshare.15125151.
